# What are the most important factors in basal cell carcinoma follow‐up care? The perspective of patients

**DOI:** 10.1002/ski2.10

**Published:** 2020-12-08

**Authors:** S. van Egmond, M. Lugtenberg, E. C. Noels, M. Wakkee, L. M. Hollestein

**Affiliations:** ^1^ Department of Dermatology Erasmus MC Cancer Institute Rotterdam The Netherlands

## Dear Editor,

Basal cell carcinoma (BCC) is the most common type of (skin)cancer worldwide in Caucasians and its incidence is still rising.^1^ The high incidence of BCC causes substantial burden on healthcare systems. This demands resources to be used efficiently, depending on the healthcare system, for example, by de‐adopting low‐value care or substituting low‐risk skin cancer care to primary care.^2^, ^3^ Aside from being efficient, care should be tailored to the needs and values of patients (i.e., patient‐centred care).^4^ Insight into the patient perspective is therefore crucial.

Previous qualitative studies among patients with BCC revealed that they prefer a physician who takes them seriously and communicates well, to receive all relevant information including a proper explanation and to be seen by the same physician each time.^2^, ^5^ In addition, they value several disease‐specific factors such as a short waiting period for the best available treatment and regular follow‐up care including a full body skin examination in order to reduce their fear and to detect new tumours early.^2^, ^5^

Although an overview of the needs of patients with BCC is informative, qualitative research does not allow us to draw conclusions on the relative importance of each of these factors to patients. For dermatologists to be able to effectively tailor their follow‐up care to the needs and preferences of patients, it is useful to assess which factors are considered most important to them. The aim of this study was therefore to determine the relative importance of factors regarding follow‐up care to patients with BCC.

A ranking list questionnaire was developed (see Data S1), based on the needs of patients elicited from previous qualitative studies.^2^, ^5^ The list consisted of five items regarding the patient‐physician relationship, five disease‐specific items and two external items (Table 1). One hundred and one consecutive BCC patients from the department of Dermatology Erasmus Medical Center (Rotterdam, The Netherlands) were asked to participate following their outpatient clinic visit with a physician for their BCC (diagnostic or follow‐up visit). After providing written informed consent, participants ranked the items from 1 (most important) to 12 (least important). The items were subsequently aggregated to include patient‐physician relationship factors (items 1,2,4,5,7), disease‐specific factors (items 3,6,8,9,10) and external factors (items 11,12). To test the differences between groups of factors, the Wilcoxon signed‐rank test was used in SPSS v24. The highest ranked group of factors was compared to the second highest group and the second highest group was tested for statistically significant difference to the third group. A *p*‐value <0.05 was considered statistically significant.

**TABLE 1:** BCC follow‐up care needs, ranked by 101 BCC patients (lower median equals more important)

	Ranking median (IQR)
Age (years)	68 (58–75)
Male	45%
Items^a^	
1. Explanation of the seriousness of skin cancer	3 (2–5)
2. Feeling that the physician listens well to the patient	4 (1–6)
3. Full skin examination during follow‐up appointment	4 (2–7)
4. Being seen by the same physician	5 (2–9)
5. Explanation of the follow‐up procedure and self‐examination of the skin	5 (3–8)
6. Early detection of skin cancer	6 (3–10)
7. Type of care provider (DE, GP, NP)	7 (5–8)
8. Side effects of skin cancer treatment	7 (5–9)
9. Frequency of follow‐up screening interval	7 (5–9)
10. Duration of the follow‐up appointment (5–20 min)	9 (6–10)
11. Costs of follow‐up care	11 (9–12)
12. Travel costs and/or travel time	11 (10–12)
Aggregated items	
Items regarding patient‐physician relationship (1, 2, 4, 5, 7)	5 (3–6)
Reference	
Items regarding disease‐specific factors (3, 6, 8, 9, 10)	7 (5–7)
Z‐score compared to patient‐physician relationship	4.5 (*p* < 0.001)
Items regarding external factors (11, 12)	11 (9.5–11.5)
Z‐score compared to disease‐specific factors	7.9 (*p* < 0.001)

Abbreviations: BCC, basal cell carcinoma; DE, dermatologists; GP, general practitioner; IQR, interquartile range; NP, nurse practitioners.

^a^ Items are ordered based on ranking score.

All of the 101 approached BCC patients completed the questionnaire (100% response rate). About one‐third of patients were diagnosed with BCC for the first time, the median age was 66 years and 56% were female. They scored patient–physician related factors as most important, with ‘explanation of the seriousness of the skin cancer’ as the most important factor regarding BCC care (Table 1). The second most important factor to patients is the ‘feeling that the physician listens well to the patient’. Patients ranked patient‐physician related factors higher than disease‐specific factors (*p* < 0.001). Of the disease‐specific factors they ranked ‘full skin examination during follow‐up appointment’ and secondly ‘early detection of skin cancer’ as most important. The external factors (costs and travel time) were considered least important (compared to disease‐specific factors; *p* < 0.001).

Whereas physicians traditionally tend to focus on disease‐oriented aspects and outcomes,^6^ this study highlights the importance of patient‐centred aspects of care to BCC patients. Particularly explanation of the seriousness of the skin cancer and the feeling that the physician listens well were considered important to patients. This is consistent with previous studies showing that physicians' interpersonal skills largely determine patient satisfaction.^7^ To facilitate physician‐patient communication, training programmes for physicians which include active, practice‐oriented strategies have been proven to be effective.^8^ Of the disease‐specific factors, patients ranked a full body skin examination as most important followed by early detection of skin cancer. Although dermatologists perform routinely full body skin examinations more often than GPs and internists (81% vs. 60% and 56% respectively), there is still room for improvement.^9^

A limitation of the current study is that only patients from a single university medical centre were included. However, the patients’ characteristics of our sample correspond well to those of the average BCC patients, which Increases the generalisability of our results.

In conclusion, findings from this study emphasize the importance of integrating patient‐physician relationship factors with traditional medically orientated aspects of BCC care. This is especially relevant because increased patient satisfaction results in increased compliance and subsequently improved health outcomes.^10^ Results of this study are currently used in a discrete choice experiment to determine which trade‐offs stakeholders are willing to make to integrate these aspects in skin cancer care.

## CONFLICT OF INTEREST

None declared.

## ETHICAL APPROVAL

Ethical approval for this study was obtained from the Medical Ethical committee of the Erasmus MC (MEC‐2014‐374).

## Supporting information

Supplementary Material
