# Supplementary material for: What are the most important factors in basal cell carcinoma follow‐up care? The perspective of patients
Source: Skin Health Dis. 2020 Dec 8;1(1):e10. doi: 10.1002/ski2.10 (PMC9060117; doi:10.1002/ski2.10)
Supplement: Supplementary file 1 — Supplementary Material [file SKI2-1-e10-s001.docx]

**Explanatory notes: completion of ranking lists**

On the following page a ranking list is presented with an explanation of the topics to be ranked. The ranking list contains 12 topics that may influence your preferences regarding skin cancer follow-up care. Could you please indicate which of the topics listed are most important to you by numbering these topics from 1-12 in the order of importance for your choice? Here 1 is the most important and/or has the most influence and 12 is the least important and/or has the least influence. Each number may only be used once.

**Topics**

- **Feeling that the physician listens well to the patient**
- **Frequency of follow-up screening interval**: The number of contact moments for follow-up checks within a certain period of time, in other words, whether you have many or few contact moments with the practitioner.
- **Side effects of skin cancer treatment**: The side effects of the treatment may differ and may include, for instance, itching, redness, pain, inflammation and a bad healing wound.
- **Costs of follow-up care**: These costs are at your deductible excess. The deductible amount can vary from €375 to €875.
- **Travel costs and/or travel time:** Some treatments are not carried out everywhere, for example Mohs’ micrographic surgery. As a result, you may have to travel a little further in order to receive the treatment.
- **Being seen by the same physician**: After the treatment, you may be seen by another physician or by the same person who treated you.
- **Duration of the follow-up appointment (5-20 minutes)**: A contact moment with the physician can vary from 5 to 20 minutes.
- **Type of care provider**: The follow-up check can be carried out by different types of practitioners: a dermatologist, a nurse specialist or a general practitioner.
- **Explanation of the seriousness of skin cancer**
- **Explanation of the follow-up procedure and self-examination of the skin**
- **Full skin examination during follow-up appointment**: Whether the physician checks the treated skin and the sun exposed areas, or whether the full body is examined.
- **Early detection of skin cancer**: With more frequent check-ups it is expected that the skin cancer can be found sooner. It makes little or no difference to the outcome whether the skin cancer is found months earlier or later.

**Ranking list: Preferences regarding skin cancer follow-up care**

| Rank | Topics that may influence your preferences  regarding skin cancer follow-up care |
| --- | --- |
|  | Feeling that the physician listens well to the patient |
|  | Frequency of follow-up screening interval |
|  | Side effects of skin cancer treatment |
|  | Costs of follow-up care |
|  | Travel costs and/or travel time |
|  | Being seen by the same physician |
|  | Duration of the follow-up appointment (5-20 minutes) |
|  | Type of care provider |
|  | Explanation of the seriousness of skin cancer |
|  | Explanation of the follow-up procedure and self-examination of the skin |
|  | Full skin examination during follow-up appointment |
|  | Early detection of skin cancer |
